# Predicting the Downward and Surface Influence of the February 2018 and January 2019 Sudden Stratospheric Warming Events in Subseasonal to Seasonal (S2S) Models

**DOI:** 10.1029/2019JD031919

**Published:** 2020-01-22

**Authors:** Jian Rao, Chaim I. Garfinkel, Ian P. White

**Affiliations:** ^1^ Fredy & Nadine Herrmann Institute of Earth Sciences The Hebrew University of Jerusalem Jerusalem Israel; ^2^ Key Laboratory of Meteorological Disaster, Ministry of Education/Joint International Research Laboratory of Climate and Environment Change/Collaborative Innovation Center on Forecast and Evaluation of Meteorological Disasters Nanjing University of Information Science and Technology Nanjing China

**Keywords:** sudden stratospheric warming (SSW), downward propagation, predictability, subseasonal to seasonal (S2S)

## Abstract

Using the real‐time predictions from 11 models, this study analyzes the prediction of the downward propagation and surface impact of the 2018 and 2019 sudden stratospheric warmings (SSWs). These two SSWs differed both in their morphology types (2018: split; 2019: displacement followed by split) and magnitudes (the former being stronger). With a large sample size (>2,200) of multimodel ensemble forecasts, it is revealed that the strength of the SSW is more important than the vortex morphology in determining the magnitude of its downward impact, with strong SSWs more likely to propagate downward than weak SSWs. Therefore, based on the probabilistic forecasts, the observed strong SSW in February 2018 was more likely to have a downward and surface impact than the January 2019 SSW. The relationship between the 10‐hPa dominant wave number and the 100‐hPa polar cap height (or the Northern Annular Mode) is weak, implying that the dominant wave number might not be the primary factor determining the downward propagation of SSWs in the two analyzed cases. Hence, the high polar cap height (or negative Northern Annular Mode) response in the lower stratosphere and troposphere following the February 2018 SSW is mainly attributed to its strong intensity rather than the split morphology. Further, the 2‐m temperature anomaly pattern following the January 2019 SSW is not forecasted due to its weak downward propagation, whereas the 2‐m temperature in North Eurasia, Middle East, south China, and eastern United States could be forecasted for the downward propagating February 2018 SSW. However, regional rainfall anomalies are poorly forecasted (both in a deterministic and probabilistic sense) for both SSWs.

## Key Points

- Better prediction of an SSW does not necessarily denote better prediction of the downward coupling or the following near‐surface impact
- The T2m in North Eurasia, Middle East, south China, and eastern United States is more difficult to forecast for the 2019 SSW with weak downward propagation
- The rainfall anomalies in some regions are poorly forecasted both in a deterministic and probabilistic sense for (non)downward propagating SSWs

## 1 Introduction

One of most extreme phenomena in the climate system is a sudden stratospheric warming (SSW) event. During a major SSW event, the temperature in the Arctic stratosphere increases by tens of kelvins, accompanied by the reversal of westerly winds in the circumpolar region to easterly winds by thermal wind balance (Charlton & Polvani, 2007; Rao, Garfinkel, et al., 2019, Rao, Ren, Chen, Liu, Yu, & Yang, 2019). Midwinter SSWs occur 6 to 7 times every decade (Butler et al., 2015; Charlton & Polvani, 2007; Hu et al., 2014; Rao et al., 2018). During the occurrence of SSW events, the Northern Annular Mode (NAM) in the stratosphere shifts toward its negative phase on average, and the negative NAM signals shows a downward propagation in the following weeks (Baldwin et al., 2003; Ren & Cai, 2007). Because the stratosphere usually has a longer memory than the troposphere, downward signals from the stratosphere might extend the predictability of surface weather (Sigmond et al., 2013; Tripathi et al., 2015, 2016). For example, after the SSW onset, anomalous cold air outbreaks on a continental scale are more likely to occur (Garfinkel et al., 2017; Karpechko, 2018; Kolstad et al., 2010; Thompson et al., 2002; Yu et al., 2015).

Since the initiation of the subseasonal to seasonal (S2S) prediction project in 2013 jointly supported by World Climate Research Program, World Weather Research Program, and The Observing System Research and Predictability Experiment, two major SSWs have been observed, one in mid‐February 2018 (Karpechko et al., 2018; Rao et al., 2018, Rao, Ren, Chen, Liu, Yu, Hu, et al., 2019) and the other in early January 2019 (Rao, Garfinkel, et al., 2019). The February 2018 SSW was a vortex split event, while the January 2019 SSW was a mixed‐type event (i.e., displacement followed by split). It has been reported that ~50% of the so‐called “split vortex” SSWs are also primarily initialized by wave number‐1 to displace the polar vortex prior to its splitting (e.g., Barriopedro & Calvo, 2014). Rao et al. (2018) use the China Meteorological Administration–Beijing Climate Center (CMA‐BCC) forecast system model to study the predictability of the February 2018 SSW. The January 2019 SSW event and its predictability are also explored (Rao, Garfinkel, et al., 2019) by using the real‐time predictions from 11 different S2S models (Vitart et al., 2017). However, the prediction of downward impacts of the 2019 SSW event on the troposphere and surface has yet (to our knowledge) to be explored using the S2S data set, although Karpechko et al. (2018) document the downward propagation of stratospheric signals related to the February 2018 SSW as well as the near‐surface climate anomalies in the ensemble mean of several S2S models.

Not all SSWs are followed by the persistent negative NAM in the troposphere or exhibit detectable impacts on the tropospheric circulation (Mitchell et al., 2013; Nakagawa & Yamazaki, 2006; Seviour et al., 2013; Sigmond et al., 2013). Based on this fact, SSW events are classified as either downward propagating or nondownward propagating events (Jucker, 2016; Karpechko et al., 2017; Kodera et al., 2016; Runde et al., 2016). The factors that may control the (non)downward propagation of SSW events include the dominant wave number of the preceding enhanced upward wave flux (Nakagawa & Yamazaki, 2006) or the SSW displacement/split type (Huang et al., 2018; Mitchell et al., 2013; Seviour et al., 2013), the strength of the upward wave forcing that precedes the SSW (White et al., 2019), the propagation of the initial circulation anomalies from the upper to lower stratosphere and the subsequent duration of lower stratospheric anomalies (Black & McDaniel, 2004; Hitchcock et al., 2013; Maycock & Hitchcock, 2015), absorption/reflection of planetary waves in the stratosphere following SSWs (Kodera et al., 2016), and preexisting tropospheric circulation conditions (Gerber et al., 2009; Hitchcock & Simpson, 2014). The relative importance of these factors is unclear, however. For example, although some studies find that the tropospheric responses are more likely to follow wave‐2 SSWs or split SSWs than wave‐1 SSWs or displacement SSWs (Mitchell et al., 2013; Nakagawa & Yamazaki, 2006; Seviour et al., 2013), other studies find little difference between the near‐surface impacts of wave‐1 and wave‐2 SSWs or between displacement and split SSWs (Maycock & Hitchcock, 2015; White et al., 2019).

In addition, it is not easy to separate the impact of the tropospheric state prior to the SSW onset on the ensuing tropospheric response (Black & McDaniel, 2004; Hitchcock & Simpson, 2014), especially if the initial tropospheric forcing responsible for the onset of the SSW is persistent and strong. For example, White et al. (2019) find that approximately 30% (60%) of SSWs are preceded by extreme episodes of wave activity in the lower troposphere (stratosphere), and the composite tropospheric wave activity is much larger preceding downward propagating SSWs compared to nondownward propagating SSWs. This enhanced wave activity is associated with zonal asymmetries which may be favorable for a downward propagation of SSW anomalies. In particular, in the model runs considered, they found that approximately two thirds of SSWs preceded by extreme tropospheric wave activity propagate downward to the troposphere.

**Figure 1:**
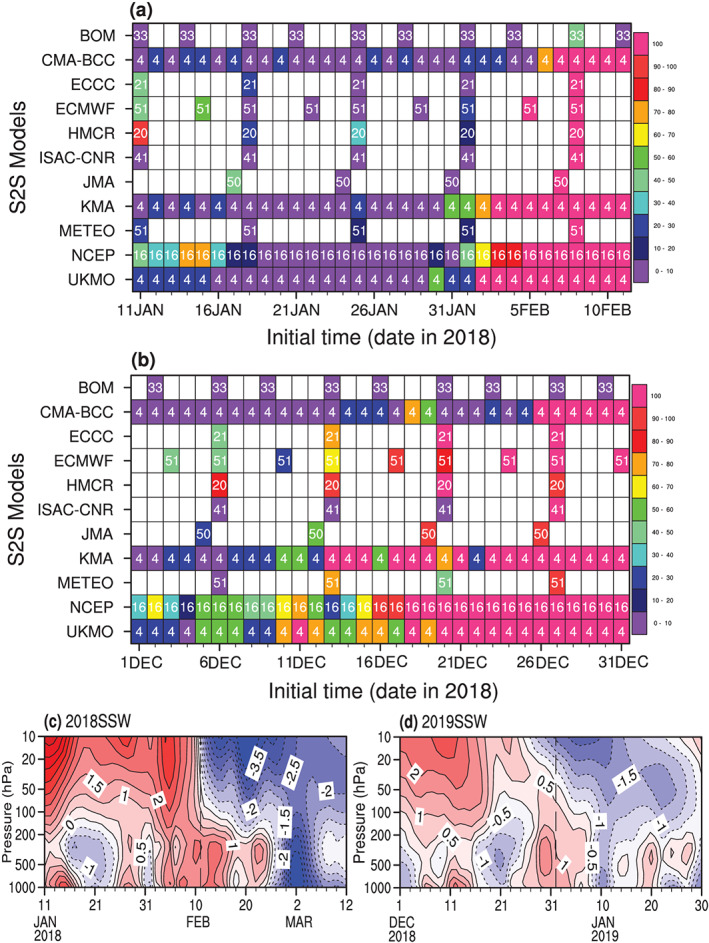
Distribution of the real‐time prediction ensemble member number (the number in each grid cell) for each model and each initialization from (a) 11 January–11 February (2,550 in total) and (b) 1–31 December 2018 (2,356 in total). The color shading in each grid cell denotes the SSW hit ratio (units: %) of the ensemble members that forecast the reversal of the zonal mean zonal wind at 60°N and 10 hPa near 11 February 2018 (top) and near 1 January 2019 (bottom), respectively, with a two‐day maximum error in timing allowed. Since the circulation data for the HMCR model at 10 hPa are unavailable, the zonal mean zonal wind at 50 hPa is used to calculate the SSW hit ratio. The blank grid denotes that no real‐time predictions were initialized on the specific day for the corresponding model. (c and d) The evolution of the NAM index for the February 2018 and January 2019 SSWs from the reanalysis (contour interval: 0.5). Figure 1b is replicated from Figure 1 in Rao, Garfinkel, et al. (2019) for an easy comparison between February 2018 and January 2019 SSW forecasts.

The real‐time predictions on the S2S time scale for the 2017/2018 and 2018/2019 winters are available from 11 S2S forecast models participating in the S2S project. Although the prediction, downward propagation, and surface impacts of the 2018 SSW using the multimodel ensemble mean (Karpechko et al., 2018) or just one model (Rao et al., 2018) have been reported recently, downward and near‐surface impacts of the 2019 SSW in the 11 forecast models, as well as the multimodel ensemble spreads for the two SSWs, have not been explored to our knowledge. Furthermore, the difference in observed downward propagation between the 2019 and 2018 events (as shown below) motivates the question: (1) what controls the magnitude of the downward impact of SSWs on the surface from a statistical perspective? We will also try to answer the additional following targeted questions: (2) what is the difference between the two SSWs in term of their intensity, type, and the skill at which they can be forecasted? (3) Is there any negative NAM‐like response in the near surface? If so, to what extent does the multimodel ensemble forecast such surface anomalies? With the forecast size increasing to more than 2,200 by incorporating all initializations within one month from all S2S models (Figures 1a and 1b), a more robust relationship can be established between the SSW and its surface influence.

The structure of the paper is organized as follow. Following this section, the S2S models and methods are introduced in section 2. Section 3 reviews the predictability of two SSW events in 11 S2S models. Prediction of the lower stratospheric responses to SSWs, both deterministic and probabilistic, is shown in section 4, followed by the surface impact of SSWs in the multiple S2S model ensemble in section 5. Finally, a summary and discussion are provided in section 6.

## 2 S2S Data and Methodology

### 2.1 The S2S Models and Real‐Time Predictions

All of the S2S real‐time forecasts initialized less than a month before the February 2018 and January 2019 SSW events from all of the 11 S2S models are used in our study. Names of the 11 S2S models are BOM (Australian Bureau of Meteorology), CMA‐BCC (China Meteorological Administration, National Climate Centre), ECCC (Environment and Climate Change Canada), ECMWF (European Centre for Medium‐range Weather Forecasts), HMCR (Hydro‐Meteorological Centre of Russia), ISAC‐CNR (Institute of Atmospheric Sciences and Climate, National Research Council of Italy), JMA (Japan Meteorological Agency), KMA (Korea Meteorological Administration), METEO (Météo‐France/Centre National de Recherche Meteorologiques), NCEP (National Centers for Environmental Prediction), and UKMO (United Kingdom Meteorological Office). Figure 1 shows all the available initializations for the 11 models with the ensemble member number filled in the grid if there are forecasts initialized on the date for the model. The real‐time predictions from the 11 S2S models are stored by the ECMWF (https://confluence.ecmwf.int/display/S2S). The ensemble member numbers, initialization dates, and initialization frequencies of the real‐time forecasts differ among the models. The forecast initialization time is different but the integration length exceeds a month for all models. The ensemble member number for a particular initialization ranges from 4 (CMA‐BCC, KMA, UKMO), to 16 (NCEP), 20 (HMCR), 21 (ECCC), 33 (BOM), 41 (ISAC‐CNR), 50 (JMA), and 51 (ECMWF, METEO). The frequencies of the forecast initializations are also different among models: weekly (ECCC, HMCR, ISAC‐CNR, JMA, METEO), twice weekly (BOM, ECMWF), and daily (CMA‐BCC, KMA, NCEP, UKMO). The integration time is also different: 62 days in BOM, 60 days in CMA‐BCC, 32 days in ECCC, 46 days in ECMWF, 61 days in HMCR, 32 days in ISAC‐CNR, 33 days in JMA, 60 days in KMA, 61 days in METEO, 44 days in NCEP, and 60 days in UKMO. More details about the model resolutions, output requirements, and model components can be found in Jie et al. (2017), Vitart et al. (2017), and references therein. All forecasts are interpolated to 2.5° × 2.5° (longitude × latitude) horizontal resolution, which is identical to the NCEP/NCAR reanalysis. The NCEP/NCAR reanalysis (Kalnay et al., 1996), both daily and monthly, are used in the study as the reference. This reanalysis is somewhat uncertain in the presatellite era, but it is as reliable in the postsatellite era as other modern reanalyses (Ayarzagüena et al., 2019).

### 2.2 Methods

**Figure 2:**
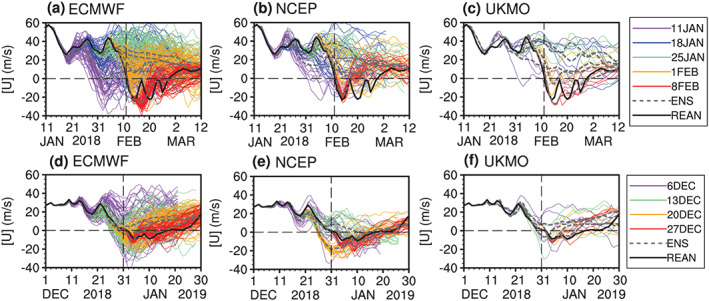
(a–c) Evolution of the zonal mean zonal wind at 60°N and 10 hPa in all ensemble members of the real‐time predictions initialized at a lead time of 31 days (purple), 24 days (blue), 17 days (green), 10 days (orange), and 3 days (red) relative to the 2018 SSW onset date (11 February 2018). (d and e) Identical to (a)–(c) but for the real‐time predictions initialized at the lead time of 25 days (purple), 18 days (green), 11 days (orange), and 4 days (red) relative to the 2019 New Year SSW onset date (1 January 2019). The thin color curves are the ensemble members, the dashed gray curves are the ensemble mean for each initialization, and the thick black curves are the reanalysis. Only three models (ECMWF, NCEP, and UKMO) are shown for succinctness. Figures 2d–2f are replicated from Figures 4d, 4j, and 4k in Rao, Garfinkel, et al. (2019) for an easy comparison between February 2018 and January 2019 SSW forecasts.

SSW events are selected based on the WMO definition (Butler et al., 2015; Charlton & Polvani, 2007) if the zonal mean zonal winds at 60°N and 10 hPa reverse from westerlies to easterlies, and the zonal mean temperature gradient between 60°N and the North Pole reverses. Accordingly, an SSW occurred on 11 February 2018 (i.e., the February 2018 SSW), and the other occurred on 31 December 2018 (i.e., the January 2019 SSW) in the reanalysis. The SSW onset dates are a little different using the zonal wind at 65°N instead, but within a three‐day window of the dates based on zonal wind at 60°N. We still use the 60°N wind for SSW identifications, because the WMO definition is one of the most widely used methods in literature. Evolutions of the zonal mean zonal winds at 60°N and 10 hPa for the two SSWs are shown in Figure 2 (black). Comparing the two SSWs, it can be seen that the intensity of the February 2018 SSW is stronger than that of the January 2019 SSW, but the persistence of the easterlies for each event at 10 hPa is nearly the same (~20 days). The easterly winds in the post‐SSW period contain subweekly variability for the former event, but the easterly winds evolve more steadily and gradually decrease and reverse back to westerly winds after 19 January 2019. We also use the polar cap geopotential height ([*Z*]_NP_) to track the NAM (e.g., Baldwin & Thompson, 2009). The supplement shows that results are similar if the NAM is defined using the leading empirical orthogonal function of the extratropical height anomalies at each pressure levels (Baldwin et al., 2003; more details can also be seen in Text S1 in the supporting information; Figures 1c and 1d).

The anomaly correlation coefficient (ACC) between forecasts and the reanalysis for a variable *T* is utilized to quantify model performance, $\mathrm{ACC}\left(t\right)=\frac{\sum_{i=1}^{n}w\left(i\right)\left[T_{\mathrm{FC}}\left(i,t\right)-\bar{T_{\mathrm{FC}}\left(t\right)}\right]\left[T_{\mathrm{NNR}}\left(i,t\right)-\bar{T_{\mathrm{NNR}}\left(t\right)}\right]}{\sqrt{\sum_{i=1}^{n}w\left(i\right){\left[T_{\mathrm{FC}}\left(i,t\right)-\bar{T_{\mathrm{FC}}\left(t\right)}\right]}^{2}}\sqrt{\sum_{i=1}^{n}w\left(i\right){\left[T_{\mathrm{NNR}}\left(i,t\right)-\bar{T_{\mathrm{NNR}}\left(t\right)}\right]}^{2}}}$. Here *t* denotes the forecast time on day *t* (*t* = 0 denotes the initial day), *i* is the spatial grid index, *n* is the total number of spatial grid points for a targeted region. The subscript “FC” denotes the forecast, and “NNR” denotes the NCEP/NCAR reanalysis. The overbars in the ACC formula denote spatial averages, including the *w*(*i*) (cosine of latitude) weighting, $\bar{T\left(t\right)}=\frac{\sum_{i=1}^{n}w\left(i\right)T\left(i,t\right)}{\sum_{i=1}^{n}w\left(i\right)}$.

## 3 A Brief Review on the Real‐Time Predictions of the Two Recent SSW Events

Figure 2 shows the observed or forecasted evolutions of the zonal mean zonal wind at 60°N and 10 hPa from three representative models and the reanalysis during both SSW events. Five common initializations are shown for the 2018 SSW with the lead time ranging from 3 to 31 days with a seven‐day interval between initialization times, and four for the 2019 SSW with the lead time ranging from 4 to 25 days. The predictions that first forecast the SSW onset are different for the two events. It can be seen from the ensemble mean of forecasts that the rapid deceleration of circumpolar westerly winds before the 2018 SSW is not forecasted for early initializations on 11, 18, and 25 January in the three models, but is forecasted for late initializations on 1 and 8 February, indicating that this SSW can be forecast at a lead time of at least 10 days in these models (Figures 2a–2c). In contrast, the slow deceleration of circumpolar westerly winds before the 2019 SSW can even be forecasted at a lead time of 25 days for initializations on 6 December 2018 (Figures 2d–2f). The zonal winds in other initializations are realistically reversed from westerlies to easterlies around 31 December 2018 in the three models. Hence, the SSW event in February 2018 is stronger, but also less predictable, than the one in January 2019.

The predictability of SSW events has been assessed as the ratio of the forecasts that accurately predict the SSW onset on the real onset date with a maximum two‐day error allowed (i.e., two days either side of the SSW onset date) for an initialization ensemble, *p* = *M*/*N* × 100 (units: %), where *M* is the number of forecast members that forecast the SSW around the targeted days for an initialization ensemble and *N* is the total number of the ensemble members for this initialization (see colors in Figure 1; also see Rao et al., 2018; Rao, Garfinkel, et al., 2019). Nearly all models have a higher ratio of the zonal westerly reversal around the real onset date in forecasts initialized at lead times of 10 days or more in Figure 1b than in Figure 1a. The predictive limit of the February 2018 SSW is ~10 days if we used a ratio threshold, ~20% as in Rao et al. (2018). In contrast, the predictive limit of the January 2019 SSW is ~20 days even if a stricter threshold (>20%) is used. The tropospheric precursor for the former SSW event include (but is not limited to) blocking/strong ridges over the North Pacific and Ural, which is not easy to forecast and disappears quickly in forecasts (Garfinkel et al., 2019; Rao et al., 2018). The favorable conditions for the latter SSWs include the persistent easterly quasi‐biennial oscillation, moderate El Niño, solar minimum, and MJO convection over the tropical western Pacific (Rao, Garfinkel, et al., 2019), and all of these phenomena have a (much) longer lifetime than a regular blocking high.

## 4 Predictability of Lower Stratospheric Response to SSW Events

### 4.1 Relationship Between the 10‐hPa SSW Intensity and Lower Level NAM Response

**Figure 3:**
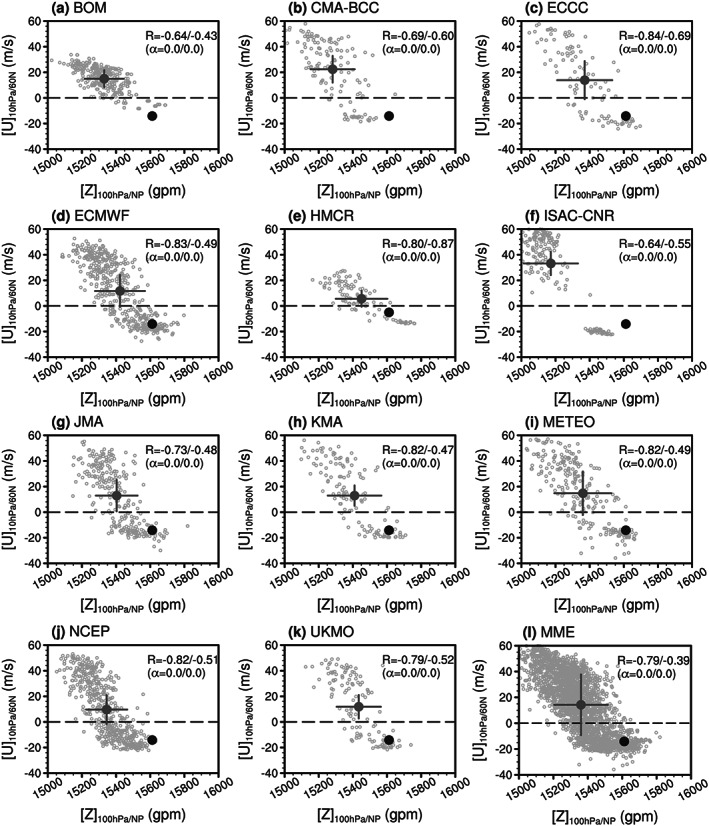
(a–k) Scatterplot of the zonal mean zonal wind at 60°N/10 hPa (ordinates) versus the area‐averaged polar (65–90°N) geopotential height at 100 hPa (abscissas) for each model. The wind is averaged from 11–15 February 2018 (with five days after the February 2018 SSW onset), and the polar geopotential height index is averaged from 11–25 February 2018 (15 days following the February 2018 SSW onset). The unfilled gray circles are the ensemble members for all initializations in each model, the filled gray circles are the average of all predictions, and the filled black circles are the reanalysis. The zonal mean zonal wind at 50 hPa is shown for the HMCR model. The correlations (*R*) between the zonal wind at 60°N and the polar geopotential height index at 100 hPa (first for all forecasts, and second for SSW‐hit forecasts) and their significance levels (*α*) also are included for each model. (l) Scatterplot of all ensemble members for all initializations in all models.

Figure 3 shows the forecast‐by‐forecast scatterplot of the zonal‐mean zonal wind at 60°N and 10 hPa on the first five days after the February 2018 SSW versus the 100‐hPa polar geopotential height averaged on the following 15 days after the SSW onset for 11 models. The observed SSW intensity is around −15 m/s, and the 15‐day mean of the 100‐hPa polar cap height is ~15,600 gpm. We mainly explore the downward impact of SSWs in the first 15 days after the SSW onset as model forecast data are only available for ~30 days for some models, and we strive to maximize data availability. As discussed in section 3, most models predict a reversal of circumpolar zonal winds mainly from forecasts initialized after 1 February 2018 (Figure 1a). The lower stratospheric NAM (polar cap height) response is positively (negatively) correlated with the circumpolar zonal winds in all forecast systems. The correlations in all forecasts and SSW‐hit forecasts between the circumpolar wind at 10 hPa and the polar cap height index at 100 hPa on the following 15 days vary with the model. Although the correlation weakens for SSW‐hit forecasts, the negative relationship between the easterlies at 10 hPa and the high polar cap height response at 100 hPa is still present in all models (Figures 3a–3k). When all forecasts and SSW‐hit forecasts (*y* < 0) from the 11 models are scattered in one plot (Figure 3l), the correlations (i.e., −0.79 in all forecasts and −0.39 in SSW‐hit forecasts) from the multimodel ensemble (MME) are also statistically significant at an ~100% confidence level (*α* = 0.0). The downward impact of SSW on the lower stratosphere can be found from all models and their MME: in a probabilistic sense strong SSWs tend to be followed by high polar cap height responses in the lower stratosphere. Identical results can also be seen in the forecast‐by‐forecast scatterplot of the zonal‐mean zonal wind at 60°N and 10 hPa versus the 100‐hPa NAM index (see Figure S4).

**Figure 4:**
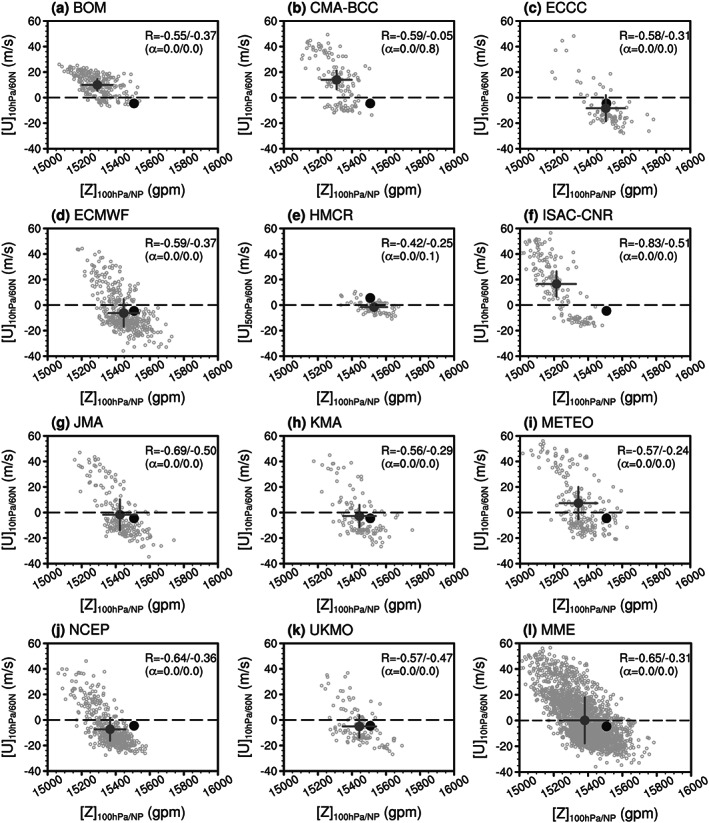
Same as in Figure 3 but for the 2019 SSW. The wind is averaged from 1–5 January 2019 (with five days after the January 2019 SSW onset), and the polar geopotential height index is averaged from 1–15 January 2019 (15 days following the January 2019 SSW onset).

Similarly, the scatterplot of the forecasted circumpolar zonal wind from 1–5 January 2019 versus the 100‐hPa polar geopotential height index averaged from 1–15 January 2019 is shown in Figure 4. The observed January 2019 SSW is relatively weak: the 10‐hPa circumpolar easterlies on the first five days is only −4 m/s, and the 100‐hPa polar cap height is only ~15,500 gpm. The weakness of the downward impacts for the January 2019 SSW are consistent with the weak intensity of the SSW itself, as model forecasts which simulate a stronger SSW than actually occurred also systematically simulate a stronger impact at 100 hPa. The negative (positive) correlation between the SSW intensity and the polar cap height (NAM) at 100 hPa can be seen among all the forecasts and SSW‐hit forecasts from each model (Figures 4 and S5). In spite of the comparable sample sizes (degrees of freedom) between the models, the correlation amplitude is largest in ISAC‐CNR (−0.83/−0.51 and 0.85/0.38; *α* = 0.0/0.0 and 0.0/0.0; Figures 4f and S5f), and much smaller in BOM (−0.55/−0.37 and 0.36/0.37; *α* = 0.0/0.0 and 0.0/0.0; Figures 4a and S5a) and METEO (−0.57/−0.24 and 0.38/0.14; *α* = 0.0/0.0 and 0.0/0.2; Figures 4i and S5i). The conclusion is also true for the MME (Figures 4l and S5l), and the correlation between 10‐hPa circumpolar zonal winds and 100‐hPa polar geopotential height (NAM) index in the following 15 days is −0.65/−0.31 (0.44/0.21). It can be seen from the MME once again that the strong easterlies (i.e., −10 m/s or stronger) that usually appear during SSWs favor the development of the 100‐hPa NAM. For moderate zonal wind between −10 and 10 m/s, the NAM index is evenly distributed between positive and negative values in the MME (Figure S5).

**Figure 5:**
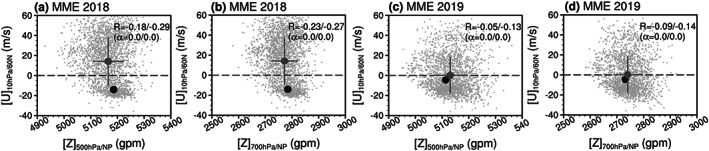
(a and b) Same as in Figure 3l but for the zonal mean zonal wind at 60°N/10 hPa (ordinates) versus the polar geopotential height index at 500 and 700 hPa (abscissas), respectively. The wind is averaged from 11–15 February 2018, and the polar geopotential height index is averaged from 16–30 February 2018. (c and d) Same as in Figure 4l but for the zonal mean zonal wind at 60°N/10 hPa (ordinates) versus the polar geopotential height index at 500 and 700 hPa (abscissas), respectively. The wind is averaged from 1–5 January 2019, and the polar geopotential height index is averaged from 6–20 January 2019. The correlations (*R*) between the zonal wind at 60°N/10 hPa and the polar geopotential height index (first for all forecasts, and second for SSW‐hit forecasts) and their significance levels (*α*) also are included for each plot.

The scatterplot of the forecasted circumpolar zonal wind from day 1 to day 5 versus the 500‐ or 700‐hPa polar geopotential height (or NAM) index averaged in the following 15 days (from day 6 to day 20) is shown in Figure 5 (or Figure S6). The general conclusion above is seldom changed, but the correlation value is much smaller for the tropospheric polar height (or NAM) than the lower stratospheric polar height (or NAM), especially for the predictions of the January 2019 SSW (cf. Figures 3l, 4l, 5, S4l, S5l, and S6). Comparing the forecasts for the two events, the correlation between the 10‐hPa zonal wind and the tropospheric polar height (NAM) is much stronger for the February 2018 SSW (Figures 5a, 5b, S6a, and S6b) than the January 2019 SSW (Figures 5c, 5d, S6c, and S6d). Based on the multimodel multiensemble mean, it is shown once again that both the 10‐hPa zonal wind and the tropospheric polar height (NAM) is better forecasted for the January 2019 SSW than the February 2018 SSW. However, the downward impact of the SSW on the ensuing tropospheric NAM is found only for the February 2018 SSW.

### 4.2 Probabilistic Predictability of the Polar Cap Height and NAM

**Figure 6:**
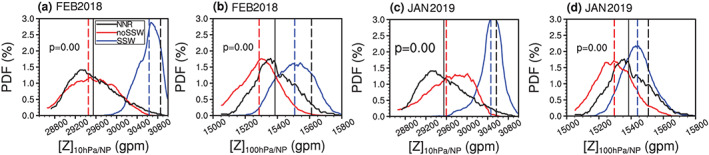
The PDF distribution of the polar geopotential height index at 10 and 100 hPa following the real SSW events. The polar geopotential height index at 10 hPa (100 hPa) is averaged within 5 (15) days after the real SSW onsets. All forecast members for all initializations from all models in Figure 1 are used to calculate the PDF of the polar geopotential height index for SSW‐missed ensemble (red) and SSW‐hit ensemble (blue), with their composite means marked by red and blue vertical dashed lines, respectively. The black curve is the climatological PDF of the wintertime (November–March) polar geopotential height index time series from the NCEP/NCAR reanalysis, with the climatology marked by a black vertical solid line. The black vertical dashed line denotes the observed 5/15‐day mean polar geopotential height value following the real SSW event in the NCEP/NCAR reanalysis. The difference in the geopotential height PDF of the SSW‐missed forecast ensemble and SSW‐hit forecast ensemble is calculated by using a nonparametric two‐sample Kolmogorov‐Smirnov test.

Figure 6 (or Figure S7) shows the probability distribution function (PDF) of the 10‐ and 100‐hPa polar cap geopotential height (or NAM) index for SSW‐missed and SSW‐hit forecasts within one month before the two SSWs from all models. All forecasts in all models are used to increase the sample size (>2,200; Figure 1) to ensure a smooth PDF distribution for the targeted index. Considering that the downward propagation of NAM index and negative NAM at 10 hPa is maximized after the SSW onset (see Figures 1c and 1d), the 10‐hPa polar height and NAM are averaged over the first five days after the observed SSW onset dates, while the 100‐hPa polar height and NAM are averaged over the first 15 days after the observed SSW onset. The climatological PDF of the polar height (NAM) index from NCEP/NCAR from 1979–2017 winters is shown in black lines as a reference. The polar height (NAM) index in the reanalysis resembles a Gaussian distribution at 10 hPa (Figures 6a, 6c, S7a, and S7c) and 100 hPa (Figures 6b, 6d, S7b, and S7d), respectively. The PDF of the polar height index at 10 hPa from SSW‐missed forecasts in the MME for the February 2018 event (i.e., the red curve in Figure 6a) peaks near the climatology (i.e., the red vertical dashed line), similar to the black line in Figure 6a. Different from the PDF for SSW‐missed forecasts, the PDF for SSW‐hit forecasts (the blue curve) significantly shifts toward large polar height values. The *p* value for the two sample distributions in forecasts for the February 2018 SSW is essentially zero (*p* ≈ 0), indicating little similarity (or significant difference) between blue and red curves according to the Kolmogorov‐Smirnov test. The composite 10‐hPa polar cap height from the SSW‐hit forecasts is ~30,600 gpm (i.e., the blue vertical dashed line in Figure 6a), smaller than the observed ~30,850 (the black vertical dashed line). The MME tends to underestimate the high polar height (or negative NAM) following the SSWs in a deterministic sense, even though the SSW onset is forecasted. The right tail of the blue curve for the 10‐hPa NAM PDF can even extend to positive values of the abscissa, that is, NAM > 0, so that even if a SSW is simulated, there is the possibility of a forecast of positive NAM responses at 10 hPa even for the February 2018 case (Figure S7a).

In the lower stratosphere, the centroid distance between the PDF of SSW‐missed forecasts and that of SSW‐hit forecasts decreases (polar height: ~0.4 km at 100 hPa versus ~1.2 km at 10 hPa in Figure 6; NAM: −0.5 at 100 hPa versus −1.5 at 10 hPa in Figure S7). It is shown once again that the 100‐hPa polar cap height (or NAM) index amplitude for the February 2018 SSW‐hit forecasts is underestimated (15,600 versus 15,500, −0.8 versus −0.5; black/blue vertical dashed lines in Figures 6b and S7b). The right tail of the NAM PDF in SSW‐hit forecasts extends far to the positive axis, so a forecasted SSW event at 10 hPa cannot ensure an ensuing negative NAM response at 100 hPa, although the ensemble mean of SSW‐hit forecasts shows a negative NAM value (Figure S7b).

Similar to the PDF of 10‐hPa polar cap height in SSW‐missed forecasts for the February 2018 SSW, the PDF peak of 10‐hPa polar cap height for the January 2019 SSW (the red curve in Figure 6c) is different from the climatology (the black curve), but it is closer to the black curve than the blue curve for SSW‐hit forecasts. The blue PDF curve for SSW‐hit forecasts in Figure 6c also shows a peak shift toward the high polar cap height (negative NAM) direction, centered at *x* = ~30,450 (i.e., the composite value of all SSW‐hit forecasts). The high polar height at 10 hPa in the reanalysis is less for the January 2019 SSW than the February 2018 SSW (~30,580 versus ~308,500 gpm), and it is also true for the 100‐hPa polar height (~15,500 versus ~15,600 gpm; Figure 6d). For the January 2019 SSW the blue curve is shifted toward high values and the *p* value is around zero at 10 hPa (Figure 6c). The forecasted 100‐hPa polar height is smaller for the January 2019 SSW than the February 2018 SSW (~15,450 versus 15,500 gpm), indicating the weak downward impact of the 2019 SSW in forecasts (Figures 6b and 6d).

### 4.3 What Plays a More Important Role for the Magnitude of the Low‐Level NAM Response: The SSW Intensity or Dominant Wave Number?

The February 2018 SSW was a wave number‐2 dominated vortex split event, while the January 2019 SSW was a mixed‐type event (i.e., wave number‐1 dominated displacement before and during its onset but split afterward). Is this difference in SSW morphology important for explaining the stronger downward impacts for the February 2018 SSW?

**Figure 7:**
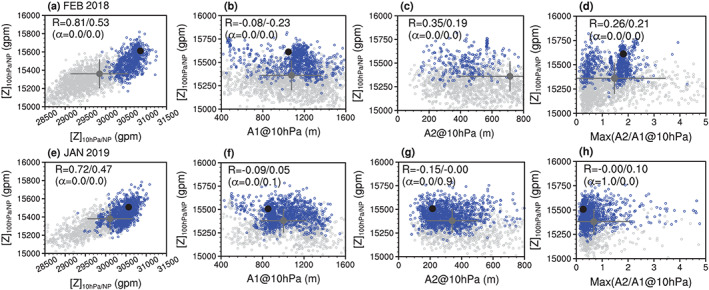
(a–d) Scatterplot of the polar geopotential height index at 10 hPa, the wave‐1 amplitude, the wave‐2 amplitude, and the maximum wave‐2/wave‐1 amplitude ratio versus the polar geopotential height index at 100 hPa (ordinates) for the February 2018 SSW. (e–h) Identical to (a)–(d) but for the 2019 New Year SSW event. All forecast members for all initializations from all models in Figure 1 are used to calculate those indices. The polar geopotential height index at 10 hPa (100 hPa) is averaged over the following 5 (15) days data after the SSW onset, the wave‐1 and wave‐2 amplitudes is averaged from day −5 to day 5, and the maximum wave‐2/wave‐1 amplitude ratio is searched from day −5 to day 5. The unfilled gray (blue) circles are all (SSW‐hit) ensemble members for all initializations in all models, the filled gray circles are the average of all predictions in all models, and the filled black circles are the reanalysis. The correlations (*R*) and their significance levels (*α*) (first for all forecasts, and second for SSW‐hit forecasts) are also printed in each plot.

Figure 7 (or Figure S8) considers the relative importance of dominant wave number and SSW magnitude in controlling downward impact to 100 hPa. Figures 7a and 7e show a scatterplot of the 10‐hPa polar‐cap geopotential height for the first five days versus 100‐hPa polar‐cap geopotential height for the first 15 days after the observed SSW onset date for all forecasts and SSW‐hit forecasts (blue circles) in all models. The 10‐hPa polar‐cap geopotential height and NAM (Figures 7a, 7e, S8a, and S8e) are used to represent the SSW intensity (Figures 3l, 4l, S4l, and S5l). In general, the lower level high height or negative NAM response is positively correlated with the SSW intensity: strong SSWs (e.g., NAM_10 hPa_ < −3) tend to propagate downward (e.g., NAM_100 hPa_ < −1) more easily than weak SSWs in forecasts (Figure S8). The correlation between the 10‐hPa SSW intensity and the 100‐hPa polar cap height (or NAM) is very stable for all forecasts (0.81 and 0.72) and SSW‐hit forecasts (0.53 and 0.47), independent of the observed case and the SSW intensity index used (Figures 3l, 4l, 7a, 7e, S4l, S5l, S8a, and S8e).

To more closely establish the relationship between dominant wave number and the possibility of downward propagation, three parameters for the 10‐hPa height averaged over 55–65°N are calculated to classify the type of SSW: the amplitude of height wave number‐1 component (A1), the amplitude of height wave number‐2 component (A2), and maximum ratio between A2 and A1 (A2/A1) from five days before to five days after the real SSW onsets. We use the A2/A1 ratio to denote the dominant wave number, which is generally much less than one for a displacement type and greater than one for a split type, but note that the A2/A1 ratio is continuous and ranges from 0 to 5 or more. For most forecasts, A2 is much smaller than A1 (Figures 7b, 7c, 7f, and 7g), and the forecasts are concentrated at max (A2/A1) <1 (Figures 7d and 7h), especially for the January 2019 case. The correlation between A1 and the 100‐hPa polar cap height (or NAM; Figures 7b, 7f, S8b, and S8f) is much smaller than the correlation between the forecasted 10‐hPa polar height (or NAM) and the 100‐hPa polar height (or NAM). It is also true for the correlation between A2 and the 100‐hPa polar height (or NAM; Figures 7c, 7g, S8c, and S8g), and that between the maximum A2/A1 ratio and the 100‐hPa polar height (or NAM; Figures 7d, 7h, S8d, and S8h). Results from all three metrics confirm that the dominant wave number is a less important factor in determining the downward impact of SSW than SSW intensity.

The relationship between the SSW strength and the downward impact is generally consistent with previous studies (e.g., Karpechko et al., 2017; Runde et al., 2016; Zhang et al., 2019; also see Table S1). The correlation between the stratospheric perturbation and the tropospheric perturbation can exceed 0.3 in two different models (Runde et al., 2016, their Figure 3), implying the importance of the SSW strength. The composite stratospheric NAM signals are much stronger in downward propagating weak stratospheric polar vortex events than in nondownward propagating events (Zhang et al., 2019, their Figure 1; Figures 1c and 1d). In addition, there are 13 downward propagating SSWs out of the observed 23 events during 1979–2014 (Karpechko et al., 2017, their Table 1): six vortex displacement events and seven vortex split events. Therefore, the vortex morphology does not predispose the downward propagation of SSW events from a statistical perspective, and as shown above SSW strength is a more important factor than morphology for the two most recent SSW events.

## 5 Predicting Surface Impact of Downward Propagating SSWs

**Figure 8:**
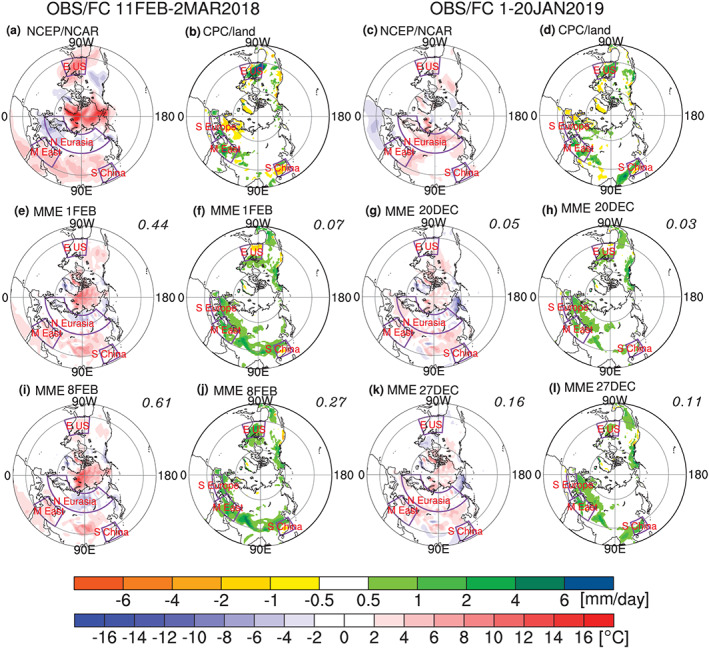
Spatial pattern of the near‐surface air temperature anomalies (blue–red/cold–warm color scale) and land precipitation anomalies (yellow–green/dry–wet color scale) in the following 20 days after the SSW onset. The NCEP/NCAR reanalysis and the CPC land precipitation are shown in the first row for reference, and the ensemble means of four initializations on 1 February, 8 February, 20 December, and 27 December are shown in the second and third rows, along with the anomaly correlation printed in the top right of each plot. The surface anomalies after the February 2018 SSW are shown in the two columns on the left, and the surface anomalies after the 2019 New Year SSW are shown on the right. The surface anomalies in four areas are marked with purple boxes for 2‐m air temperature (Southern Europe, Middle East, south China, and eastern United States) and land precipitation (Northern Eurasia, Middle East, south China, and eastern United States), respectively. Figures 8a, 8e, and 8i are similar to Figures 4b, 4d, and 4f in Karpechko et al. (2018), which are updated for an easy comparison between February 2018 and January 2019 SSW forecasts. Note that the MME is based on 11 models in this study, while Karpechko et al. (2018) use 9 models.

Figure 8 shows the 2‐m temperature and precipitation anomalies in the following 20 days after the SSW onset in four initializations: 1 February, 8 February, 20 December, and 27 December 2018. The first two initializations predict the 2018 SSW at a lead time of 10 and 3 days, and the last two initializations predict the 2019 SSW at a lead time of 11 and 4 days. Comparing the two SSW events, only the former has a downward propagating impact: North Eurasia is ~4 °C colder than normal, while the Middle East, south China, and eastern United States are 2–4 °C warmer (Figure 8a; note that warming over the eastern United States is atypical for the period after SSW; Kolstad et al., 2010; Thompson et al., 2002). The MME can well forecast cooling over north Eurasia for initializations on 1 and 8 February, although the warm anomalies in Middle East, south China, and eastern United States are underestimated (Figures 8e and 8i). The ACC for 2‐m temperature is 0.44 for the 1 February initialization and increases to 0.61 for the 8 February initialization. In contrast, the predictability of rainfall anomalies in the extratropical land of Northern Hemisphere is low, especially for the 1 February initialization (Figures 8b, 8f, and 8j). Anomalously more rainfall appears in eastern United States, Southern Europe, and the Middle East, and less rainfall appears in Northern Europe and south China (Figure 8b). The dry south China and wet Southern Europe–Middle East are well forecasted (Figures 8f and 8j), but not the precipitation impacts over Northern Europe. Wet conditions over eastern United States are forecasted only in the 8 February initialization (Figure 8j).

In contrast, the January 2019 SSW was a nondownward propagating event, and consistent with this the surface impacts typically associated with an SSW are not present in the forecasts. The 2‐m temperature in North Eurasia is anomalously warm (opposite to the anomalies typically associated with SSW; Thompson et al., 2002), while temperature anomalies in other regions are quite weak (Figure 8c). The predictability of warm anomalies in North Eurasia is very low: the forecasted temperature anomalies in this region are quite weak (<2 °C), because the nondownward propagating SSW in the MME cannot exert impacts on the near surface (Figures 8g and 8k). The observed warm anomalies in North Eurasia are likely related to tropospheric internal variability rather than the stratospheric impact. Similar to the near‐surface temperature, the regional rainfall anomalies are poorly forecasted by the two initializations, with quite low ACCs (i.e., 0.03, 0.11). The limited predictive skill for positive rainfall anomalies in Middle East, south China, and eastern United States (Figures 8d, 8h, and 8l) might also be related to tropical forcings (e.g., MJO and El Niño), although this is beyond the scope of this paper.

**Figure 9:**
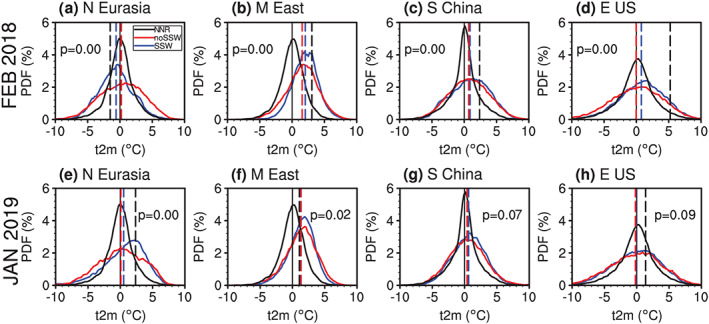
The PDF distribution of the 2‐m temperature anomalies in the following 20 days after the real SSW onset in four regions, including (a and e) North Eurasia (50–70°N, 0–140°E), (b and f) Middle East (30–45°N, 30–60°E), (c and g) south China (21–33°N, 108–123°E), and (d and h) eastern United States (30–45°N, 70–95°W). All forecast members for all initializations from all models in Figure 1 are used to calculate the PDF of the 2‐m temperature anomalies for SSW‐missed ensemble (red) and SSW‐hit ensemble (blue), with their composite means marked by red and blue vertical dashed lines, respectively. The black curve is the climatological PDF of the wintertime 2‐m temperature anomaly time series from the NCEP/NCAR reanalysis, with the mean at zero marked by a black vertical solid line. The black vertical dashed line denotes the observed 20‐day mean 2‐m temperature anomaly values following the real SSW event. The difference in the 2‐m temperature PDF of the SSW‐missed forecast ensemble and SSW‐hit forecast ensemble is calculated by using a nonparametric two‐sample Kolmogorov‐Smirnov test.

Figure 9 displays the PDF of the 2‐m temperature anomalies as shown in Figure 8a but from all forecasts and all models for four regions. The PDFs of temperature anomalies in SSW‐missed forecasts (initialized on different days) with data available during the focused periods (the red curve) and that in SSW‐hit forecasts (the blue curve) are separately shown. Specifically, the PDF of North Eurasian temperature moves toward colder values (cf. red and blue curves), indicating that downward propagating SSWs, on average, are followed by a cold North Eurasia (Figure 9a). In contrast, the temperature PDF in the Middle East, south China, and eastern United States moves toward warmer temperatures (Figures 9b–9d). These temperature impacts are similar to those found by Thompson et al. (2002) in a composite of many weak‐vortex events. For the nondownward propagating SSW in January 2019, the PDF of temperature anomalies in SSW‐missed forecasts and that in SSW‐hit forecasts are nearly indistinguishable, especially for Middle East, south China, and eastern United States (Figures 9e–9h).

**Figure 10:**
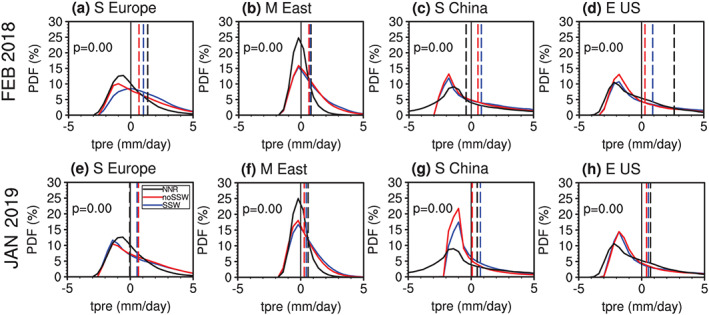
Same as in Figure 9 but for the PDF distribution of the land precipitation anomalies in the following 20 days after the real SSW onset in four regions, including (a and e) Southern Europe (35–45°N, 0–30°E), (b and f) Middle East (30–45°N, 30–60°E), (c and g) south China (21–33°N, 108–123°E), and (d and h) eastern United States (30–45°N, 70–95°W). Note that the four regions checked for precipitation are not exactly the same as for the 2‐m temperature.

The PDF of surface rainfall anomalies of all forecasts in the MME is shown in Figure 10. The PDF of the Southern Europe and eastern United States rainfall in SSW‐hit forecasts shifts toward positive anomalies relative to the PDF of SSW‐missed forecasts for the 2018 SSW (Figures 10a and 10d), indicating that the positive precipitation anomalies in those two regions in observations are related to the downward propagating SSW. The models demonstrate neither deterministic nor probabilistic skill in forecasting rainfall in the other two regions using SSW events, whether downward propagating (Figures 10b and 10c) or not (Figures 10f and 10g). The composite rainfall amounts of SSW‐missed forecasts and that of SSW‐hit forecasts for the nondownward propagating SSW are nearly the same in Southern Europe, Middle East, and eastern United States (Figures 10e, 10f, and 10h), although the large sample size gives a low *p* value for the rainfall PDFs. While the stratosphere can modulate the tropospheric circulation, rainfall is also related to tropospheric convection, divergence, and moisture availability. The prediction of rainfall is apparently much more difficult than the prediction of circulation and temperature. This is consistent with Figure 8: ACC for 2‐m temperature is much larger than ACCs for land rainfall.

## 6 Summary and Discussion

This study analyzes the real‐time prediction of downward propagation and surface impact of two recent SSWs in S2S models. The relationship between the SSW intensity and the lower level response and that between the dominant wave number and 100‐hPa polar cap height (or NAM) response is explored in a large ensemble of >2,200 forecasts. Deterministic and probabilistic predictability of low‐level NAM‐like response for the February 2018 and January 2019 SSWs are analyzed in this study. This study builds on Rao, Garfinkel, et al. (2019) who compared the predictability of the SSW in January 2019 in these same S2S models. The predictable limit for the January 2019 SSW has been reported to be longer than the February 2018 SSW (also see Figure 1), which might be related to different precursors and their persistence time scale. The predictable limit for the January 2019 SSW is more than three weeks in most S2S models, but only around 10 days in most models for the February 2018 event. In addition, the intensity of the January 2019 SSW is much weaker than the February 2018 SSW. The main conclusions in this study are as follows.

1. Better prediction of an SSW does not denote better prediction of the near‐surface impact if the SSW is a nondownward propagating event, such as the January 2019 SSW. In contrast, shorter predictability of the February 2018 SSW does not correspond to poor prediction of the surface because this SSW is a downward propagating event (Figure 8).
2. The 10‐hPa SSW intensity measured by the first five‐day mean zonal wind in the circumpolar region or 10‐hPa polar cap height (i.e., the NAM) is positively correlated with the lower stratospheric and tropospheric response (Figures 3, 4, 5). Namely, a strong SSW tends to propagate downward more easily than a weak SSW in the MME. The PDF of the 100‐hPa polar cap height (NAM) from SSW‐hit forecasts in the MME shifts toward the high (negative) value direction relative to that from SSW‐missed forecasts in the MME (Figure 6). Therefore, from the perspective of probabilistic prediction, the observed strong SSW in February 2018 is more likely to have a downward impact, as compared to the observed event in January 2019.
3. The relationship between the wave‐2/wave‐1 amplitude (ratio) and 100‐hPa polar cap height (or NAM) is much weaker than that between the SSW intensity and 100‐hPa polar cap height (or NAM). This may imply that the dominant wave number is not the primary factor determining the downward propagation of SSWs (Figure 7). No evidence in our study indicates that wave number‐2 vortex perturbations are more likely to propagate downward than wave number‐1 vortex perturbations. The negative NAM response in the lower stratosphere and troposphere following the February 2018 SSW is associated with its strong intensity rather than the split morphology.
4. In the following 20 days after the February 2018 SSW onset, the predicted near‐surface air temperature anomaly pattern is highly consistent with the reanalysis in the MME forecasts initialized on 1 February and especially on 8 February. In contrast, the 2‐m air temperature anomaly pattern following the January 2019 SSW is poorly forecast (Figure 8). The 2‐m air temperature in North Eurasia, Middle East, and south China can be forecast for downward propagating SSWs, while it cannot be for nondownward propagating SSWs (Figure 9). Although rainfall is inherently more difficult to predict than 2‐m temperature, the regional rainfall following SSWs is more predictable in SSW‐hit forecasts than in SSW‐missed forecasts at the same initialization lead times for the 2018 downward propagating SSW. In contrast, for the nondownward propagating 2019 SSW, neither probabilistic nor deterministic skill is evident for forecasts of rainfall (Figure 10).

With a large sample size of forecasts from various ensemble members of different initialization times in different models, this study reveals that the intensity of SSWs is positively correlated with the lower level high polar cap height (or negative NAM) response. The downward propagation of SSWs is evenly and randomly distributed between wave number‐2 SSWs and wave number‐1 SSWs in the forecast MME, consistent with White et al. (2019). Based on different definitions of SSW split/displacement types and downward/nondownward impact, the number of downward propagating SSWs and especially the number of nondownward propagating SSWs is similar between split and displacement SSWs (White et al., 2019, their Table 1). Maycock and Hitchcock (2015) also do not find large differences between split and displacement types. We also revisit SSW events in the reanalysis during 1979–2014 and find that nearly half downward propagating SSWs are displacement events and the other half are split events (Karpechko et al., 2017, their Table 1; also see Table S1). Please note that the wave number‐1 SSW in this study is not necessarily a displacement event, and wave number‐2 is not necessarily a split event.

However, some previous observational and modeling studies indicate the favorable initial conditions for downward propagation of SSW signals, including the dominating wave‐2 preceding SSW onset or the so‐called SSW split type (Mitchell et al., 2013; Nakagawa & Yamazaki, 2006; Seviour et al., 2013), the absorptive (as opposed to reflective) property of stratospheric planetary waves (Kodera et al., 2016), the persistent lower stratospheric circulation anomalies (Hitchcock & Simpson, 2014; Jucker, 2016; Karpechko et al., 2017), and the preexisting tropospheric conditions (Black & McDaniel, 2004; Gerber et al., 2009; Hitchcock & Simpson, 2014; White et al., 2019) or the preexisting and persistent enhanced upward propagating planetary waves (Karpechko et al., 2017; Lehtonen & Karpechko, 2016). Because our prediction study uses the forecasts initialized on different days, and the prediction length is limited, the preexisting wave activities in the troposphere before the initialization time are not analyzed here. The enhanced wave activities are usually, although not necessarily, responsible for the SSW onset and intensity; that is, 30–35% SSWs are preceded by extreme tropospheric wave activity (Birner & Albers, 2017; de la Cámara et al., 2019; Rao, Garfinkel, et al., 2019, Rao, Ren, Chen, Liu, Yu, & Yang, 2019; White et al., 2019), and the lower stratospheric anomalies are also usually less persistent for weak SSWs. The coincidence of downward propagation of some split SSWs might be related to their large intensity, such as the February 2018 SSW.

Forecasts initialized near the downward propagating SSW onset successfully predict the near‐surface air temperatures of different regions in the first 20 days after SSW onset. Although the targeted period is different between this study (15–20 days on the subseasonal time scale) and previous studies (e.g., 10 days in Black and McDaniel (2004), one month in Karpechko et al. (2018), two months in White et al. (2019)), cooling over North Eurasia is observed in all studies following downward propagating SSWs. Forecasts initialized near the nondownward propagating SSWs might indicate less enhanced skill than forecasts initialized near the downward propagating SSWs in predicting rainfall and surface temperatures over some continental regions of the Northern Hemisphere.

This paper only focuses on the two most recent SSW cases using the S2S real‐time forecasts, and more SSW cases are still required to generalize the relationship between the SSW intensity, SSW morphology, and its downward impact in S2S models. The large S2S hindcasts might provide a possibility of further studying the SSW predictability (e.g., Domeisen et al., 2020; Karpechko, 2018; Rao, Ren, Chen, Liu, Yu, Hu, et al., 2019) and its downward impact in the future.

## Supporting information



Supporting Information S1
