# Supplementary material for: Predicting the Downward and Surface Influence of the February 2018 and January 2019 Sudden Stratospheric Warming Events in Subseasonal to Seasonal (S2S) Models
Source: J Geophys Res Atmos. 2020 Jan 22;125(2):e2019JD031919. doi: 10.1029/2019JD031919 (PMC7507786; doi:10.1029/2019JD031919)
Supplement: Supplementary file 1 — Supporting Information S1 [file JGRD-125-e2019JD031919-s001.docx]

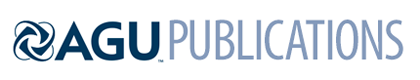


*Journal of Geophysical Research Atmospheres*

Supporting Information for

**Predicting the Downward and Surface Influence of the February 2018 and January 2019 Sudden Stratospheric Warming events in Subseasonal to Seasonal (S2S) Models**

Jian Rao^1,2^, Chaim I. Garfinkel^1^, Ian P. White^1^

^1^Fredy & Nadine Herrmann Institute of Earth Sciences, The Hebrew University of Jerusalem, Edmond J. Safra Campus, Givat Ram, Jerusalem 91904, Israel

^2^Key Laboratory of Meteorological Disaster, Ministry of Education (KLME) / Joint International Research Laboratory of Climate and Environment Change (ILCEC) / Collaborative Innovation Center on Forecast and Evaluation of Meteorological Disasters (CIC-FEMD), Nanjing University of Information Science & Technology, Nanjing 210044, China

**Contents of this file**

Text S1

Table S1

Figures S1 to S8

Text S1.

The monthly geopotential height anomalies from the NCEP/NCAR reanalysis are used to calculate the leading empirical orthogonal function (EOF) in the extratropics at 10hPa after the height anomalies are weighted by the square root of cosine of latitudes. Because the grids are uniformly distributed in the latitude-longitude grid, the grid area is proportional to the cosine of the latitude (i.e., *dS*=*R*^2^cos*φdφdλ*). The covariance of height anomalies, therefore, are weighted by the grid area. The leading EOF of the extratropical height anomalies is defined as the NAM pattern (e.g., Baldwin et al. 2003). The standard deviation (*σ*) of the leading EOF time series is saved for future use. Then the daily extratropical height anomalies at 10hPa in the NCEP/NCAR reanalysis from 11 January–12 March 2018 (i.e., two months around the February 2018 SSW) and from 1 December 2018–30 January 2019 (i.e., two months around the January 2019 SSW) are projected onto the NAM pattern to calculate the daily NAM index for reference. To highlight the downward propagation of the NAM, the NAM index is divided by *σ* and non-dimensionalized. The weighted geopotential height anomalies (i.e., multiplied by $\sqrt{\cos\varphi}$) in all forecasts from all models are also projected onto the above NAM pattern to calculate the daily NAM index in models. The NAM index in all models is also normalized by *σ* and non-dimensional.

The EOF analysis on the extratropical height anomalies are performed for each pressure level separately. As an example, the pressure-time evolutions of the NAM index for the two SSWs from the reanalysis are shown in Figures 1c and 1d. Obviously, the February 2018 SSW has a stronger stratospheric NAM and a larger tropospheric impact than the January 2019 SSW. The anomaly here refers to the deviation from the 60-day running mean daily climatology over 1979–2017 in the reanalysis. Note that the total S2S hindcast data are several hundred Terabytes (TB) large, so we try to calculate the model winter climatology on each forecast day with non-exhaustive hindcasts (i.e., once a week) initialized in the northern winter during the common hindcast years (e.g., 1995–2014) for all S2S models (see Figure S1). Then the forecast day-based climatology in winter is also used to extract the forecast anomalies on different forecast days.

However, we find the anomalies relative to the reanalysis climatology are very similar to those relative to the model climatology. As an example, the NAM index at 10hPa in the 1 February 2018 initializations are shown in Figures S2 and S3 using the reanalysis and model climatology, respectively. Nearly no detectable improvement can be seen from Figures S3 to Figures S4. These model biases likely grow with the integration time, but we are interested in at most the first 60 days before the climatology bias becomes large enough to affect the radical circulation anomalies during SSWs. Since the NAM index can also be represented by the polar cap height anomalies but with the sign reversed, we use the area-averaged polar height ([*Z*]_NP_) without removal of the climatology to replace the NAM index in most figures. The NAM index based on the height anomalies with respect to the model climatology is correspondingly provided in the supporting information (Figures S4–S8).

Table S1. Onset dates of northern winter SSW events during the S2S common hindcast and forecast period 1995–2019. The SSW events are either vortex displacement (D) or vortex split (S) events, downward (with tropospheric impact) or non-downward (without tropospheric impact) propagating events. The minimum zonal winds (maximum easterlies) are also listed.

| No. | Onset date | Maximum easterlies | Tropospheric impact |
| --- | --- | --- | --- |
| 1 | 14 December 1998 (D) | -23 | Yes* |
| 2 | 25 February 1999 (S) | -17 | Yes |
| 3 | 20 March 2000 (D) | -3 | No |
| 4 | 16 February 2001 (S) | -12 | Yes |
| 5 | 30 December 2001 (D) | -2 | No |
| 6 | 18 January 2003 (S) | -2 | No |
| 7 | 7 January 2004 (D) | -14 | Yes |
| 8 | 21 January 2006 (D) | -25 | Yes |
| 9 | 24 February 2007 (D) | -8 | No |
| 10 | 22 February 2008 (D) | -14 | No |
| 11 | 24 January 2009 (S) | -29 | Yes |
| 12 | 9 February 2010 (S) | -7 | Yes |
| 13 | 24 March 2010 (D) | -2 | No |
| 14 | 6 January 2013 (S) | -13 | Yes |
| 15 | 11 February 2018 (S) | -24 | Yes |
| 16 | 1 January 2019 (D+S) | -8 | No |

Figure S1. The climatology of (a) the zonal-mean zonal wind at 60°N and 50hPa, (b) area-averaged polar geopotential height at 50hPa, and (c) area-averaged polar air temperature at 50hPa. The climatology is extracted from available hindcasts initialized in winter (December–February) during 1995–2014, as a function of the predict day. The winter climatology from the NCEP/NCAR reanalysis during 1995–2014 is also shown as the reference (black). The model climatology is shown in colors.

**Figure S2.** Evolution of the 10-hPa NAM index in all ensemble members of the real-time predictions initialized on 1 February 2018. The solid gray curves are the ensemble members, the dashed gray curves are the ensemble mean, and the thick black curves are the reanalysis. Only forecasts initialized on 1 February 2018 are shown for clarity and simplicity. The horizontal dashed lines are zero lines, and the vertical dashed lines mark the February 2018 SSW onset date. The height anomalies at 10 hPa with respect to the reanalysis climatology are used to extract the NAM index for each model.

**Figure S3.** Similar to Figure S2, but the height anomalies at 10 hPa with respect to the forecast day-based climatology are used to extract the NAM index for each model. Nearly no detectable improvement can be seen from Figures S3 to Figures S4.

Figure S4. (a–k) Scatterplot of the zonal mean zonal wind at 60°N/10hPa (ordinates) versus the NAM index at 100hPa (abscissas) for each model. The wind is averaged from 11–15 February 2018 (with five days after the February 2018 SSW onset), and the NAM index is averaged from 11–25 February 2018 (15 days following the February 2018 SSW onset). The unfilled gray circles are the ensemble members for all initializations in each model, the filled gray circles are the average of all predictions, and the filled black circles are the reanalysis. The zonal mean zonal wind at 50hPa is shown for the HMCR model. The correlations (*R*) between the zonal wind at 60°N/10hPa and the NAM index at 100hPa (first for all forecasts, and second for SSW-hit forecasts) and their significance levels (*α*) also are included for each model. (l) Scatterplot of all ensemble members for all initializations in all models.

Figure S5. As in Figure S4 but for the 2019 SSW. The wind is averaged from 1–5 January 2019 (with five days after the January 2019 SSW onset), and the NAM index is averaged from 1–15 January 2019 (15 days following the January 2019 SSW onset).

Figure S6. (a, b) As in Figure S4l, but for the zonal mean zonal wind at 60°N/10hPa (ordinates) versus the NAM at 500hPa and 700hPa (abscissas), respectively. The wind is averaged from 11–15 February 2018, and the NAM index is averaged from 16–30 February 2018. (c, d) As in Figure S5l, but for the zonal mean zonal wind at 60°N/10hPa (ordinates) versus the NAM index at 500hPa and 700hPa (abscissas), respectively. The wind is averaged from 1–5 January 2019, and the NAM index is averaged from 6–20 January 2019. The correlations (*R*) between the zonal wind at 60°N/10hPa and the NAM index (first for all forecasts, and second for SSW-hit forecasts) and their significance levels (*α*) also are included for each model.

Figure S7. The PDF distribution of the NAM index at 10hPa and 100hPa following the real SSW events. The NAM index at 10hPa (100hPa) is averaged within 5 (15) days after the real SSW onsets. All forecast members for all initializations from all models in Figure 1 are used to calculate the PDF of the NAM index for SSW-missed ensemble (red) and SSW-hit ensemble (blue), with their composite means marked by red and blue vertical dashed lines, respectively. The black curve is the climatological PDF of the wintertime (November–March) NAM index timeseries from the NCEP/NCAR reanalysis, with the mean at zero marked by a black vertical solid line. The black vertical dashed line denotes the observed 5/15-day mean NAM value following the real SSW event in the NCEP/NCAR reanalysis. The difference in the NAM PDF of the SSW-missed forecast ensemble and SSW-hit forecast ensemble is calculated by using a nonparametric two-sample Kolmogorov-Smirnov test.

Figure S8. (a–d) Scatterplot of the NAM index at 10hPa, the wave-1 amplitude, the wave-2 amplitude, and the maximum wave-2/wave-1 amplitude ratio versus the NAM index at 100hPa (ordinates) for the February 2018 SSW. (e–h) Identical to (a–d), but for the 2019 New Year SSW event. All forecast members for all initializations from all models in Figure 1 are used to calculate those indices. The NAM index at 10hPa (100hPa) is averaged over the following 5 (15) days data after the SSW onset, the wave-1 and wave-2 amplitudes is averaged from day –5 to day 5, and the maximum wave-2/wave-1 amplitude ratio is searched from day –5 to day 5. The unfilled gray (blue) circles are all (SSW-hit) ensemble members for all initializations in all models, the filled gray circles are the average of all predictions in all models, and the filled black circles are the reanalysis. The correlations (*R*) and their significance levels (*α*) (first for all forecasts, and second for SSW-hit forecasts) are also printed in each plot.
